# Responsible data sharing in a big data-driven translational research platform: lessons learned

**DOI:** 10.1186/s12911-019-1001-y

**Published:** 2019-12-30

**Authors:** S. Kalkman, M. Mostert, N. Udo-Beauvisage, J. J. van Delden, G. J. van Thiel

**Affiliations:** 1 Julius Center for Health Sciences and Primary Care, University Medical Center Utrecht, Utrecht University, Universiteitsweg 100, 3584 CG Utrecht, the Netherlands; 2 Servier Monde, 50 Rue Carnot, 92284 Suresnes, France

**Keywords:** Big data, Data sharing, Data access, Governance, Ethics

## Abstract

**Background:**

To foster responsible data sharing in health research, ethical governance complementary to the EU General Data Protection Regulation is necessary. A governance framework for Big Data-driven research platforms will at least need to consider the conditions as specified a priori for individual datasets. We aim to identify and analyze these conditions for the Innovative Medicines Initiative’s (IMI) BigData@Heart platform.

**Methods:**

We performed a unique descriptive case study into the conditions for data sharing as specified for datasets participating in BigData@Heart. Principle investigators of 56 participating databases were contacted via e-mail with the request to send any kind of documentation that possibly specified the conditions for data sharing. Documents were qualitatively reviewed for conditions pertaining to data sharing and data access.

**Results:**

Qualitative content analysis of 55 relevant documents revealed overlap on the conditions: (1) only to share health data for scientific research, (2) in anonymized/coded form, (3) after approval from a designated review committee, and while (4) observing all appropriate measures for data security and in compliance with the applicable laws and regulations.

**Conclusions:**

Despite considerable overlap, prespecified conditions give rise to challenges for data sharing. At the same time, these challenges inform our thinking about the design of an ethical governance framework for data sharing platforms. We urge current data sharing initiatives to concentrate on: (1) the scope of the research questions that may be addressed, (2) how to deal with varying levels of de-identification, (3) determining when and how review committees should come into play, (4) align what policies and regulations mean by “data sharing” and (5) how to deal with datasets that have no system in place for data sharing.

## Background

The sharing of clinical research data is increasingly viewed as a moral duty [1]. Particularly in the context of making clinical trial data widely available, editors of international medical journals have labeled data sharing a highly efficient way to advance scientific knowledge [2–4]. The combination of even larger datasets into so-called “Big Data” is considered to offer even greater benefits for science, medicine and society [5]. Several international consortia have now promised to build grand-scale, Big Data-driven translational research platforms to generate better scientific evidence regarding disease etiology, diagnosis, treatment and prognosis across various disease areas [6–8].

Despite anticipated benefits, large-scale sharing of health data is charged with ethical questions. Stakeholders have been urged to consider how to manage privacy and confidentiality issues, ensure valid informed consent, and determine who gets to decide about data access [9]. More fundamentally, new data sharing activities prompt questions about social justice and public trust [10]. To balance potential benefits and ethical considerations, data sharing platforms require guidance for the processes of interaction and decision-making. In the European Union (EU), legal norms specified for the sharing of personal data for health research, most notably those set out in the General Data Protection Regulation (GDPR) (EU 2016/679), remain open to interpretation and offer limited practical guidance to researchers [11–13]. Striking in this regard is that the GDPR itself stresses the importance of adherence to ethical standards, when broad consent is put forward as a legal basis for the processing of personal data. For example, Recital 33 of the GDPR states that data subjects should be allowed to give “consent to certain areas of scientific research when in keeping with recognised ethical standards for scientific research” [14]. In fact, the GDPR actually encourages data controllers to establish self-regulating mechanisms, such as a code of conduct. To foster responsible and sustainable data sharing in translational research platforms, *ethical* guidance and governance is therefore necessary. Here, we define governance as ‘the processes of interaction and decision-making among the different stakeholders that are involved in a collective problem that lead to the creation, reinforcement, or reproduction of social norms and institutions’ [15].

In the design of a Big Data-driven translational research platform, any form of co-created ethical governance will at least need to relate to the conditions for data sharing as specified a priori for datasets that participate in that particular platform (in the following referred to as ‘prespecified conditions’). For example, the informed consent form of a clinical trial might only allow secondary use on the basis of re-consent from the participant. Policy documents of an observational study might only allow for the issuance of aggregate data instead of individual patient data. Apart from their practical significance to specific data sharing platforms, we anticipate that such conditions will also provide us with relevant “moral wisdom” about what values and principles could be at stake.

In this explorative study, we make use of the Innovative Medicines Initiative’s (IMI) BigData@Heart platform to answer the question: What conditions regarding sharing of datasets are specified a priori by the policies of the respective studies that participate in BigData@Heart? As of March 2017, the public-private BigData@Heart consortium has started to assemble a vast array of international (mostly European) datasets including millions of patients with the ambition of creating an open-access informatics platform to foster evidence generation in the field of cardiovascular medicine [8, 16]. Investigation of BigData@Heart will generate insights into prespecified conditions for data sharing that [1] help to respect original agreements between data subjects and researchers, [2] uncover site-specific legal and ethical conditions for data sharing and [3] expose where additional efforts are needed for the development of a governance framework for international data sharing in health research. As such, our results will not only be of high value to the BigData@Heart consortium, but to any other initiative that has the ambition of establishing a Big Data-driven research platform.

## Methods

For this exploratory case study, we followed a three-step approach. First, we identified the dataset characteristics in BigData@Heart. We subsequently attempted to obtain any relevant material from the study teams in terms of ethico-legal documents that were likely to mention conditions for data sharing. Lastly, we performed a thematic content analysis of prespecified conditions, with a particular focus on overlap and divergence. As this study does not constitute human subjects research but source document research, for which materials were voluntarily provided by affiliated study teams for the particular goal of this study, no ethics approval was required.

### Dataset characteristics

At the start of BigData@Heart in March 2017, 46 datasets were listed in the Description of Action (DoA) to contribute patient-level data from more than 25 million individuals. Seven major sources of data were mentioned: (1) disease-based genetic collections (acute coronary syndrome, atrial fibrillation, heart failure) (2); disease-based collections including omics data (3); hospital-based electronic health records (EHR) data (4); population-based (consented) cohorts (5); healthy population cohorts with omics; and (6) clinical trial data. From each of the 46 datasets the following variables were extracted: name of dataset (*usually study acronym*); principal investigator (PI) and/or data custodian; organization of PI; contact details of PI and/or data custodian; type of data; study design; cohort/sample size; and countries involved.

### Retrieval of ethico-legal documents

To review the conditions under which the different data were originally collected, we asked study teams for key ethico-legal documentation: informed consent and patient information forms, data transfer agreements (DTAs) and/or (institutional/national) policy documents on data sharing. A senior study team member (JvD) contacted all PIs of the databases personally by e-mail in September 2017. When the PI of a particular database had not responded and the data manager was known, this person was contacted as well. Contacts in the Netherlands were telephoned after a reminder e-mail when there had been no response. Only if a respondent answered that there were no documents, a member of the study team (SK) would inquire about the reasons. In such cases, respondents were specifically asked about references to institutional or other relevant policy documents.

### Analysis

In cases where documents had been provided, we noted the number and type of documents that had been sent to our team. The aggregate of documents was subsequently analyzed in a qualitative manner, indicating that text was searched for any mentioning of conditions for data sharing. Iterative content analysis (indicating a going back and forth between the sources and the draft structure for organizing findings) were used to reconstruct conditions. Identified restrictions and omissions were not linked to specific datasets in the analysis since the objective was to obtain more general insights into the content and variation of conditions in such a project. Lastly, identified conditions were thematically categorized [17].

## Results

### Datasets and documents

Inquiry within the BigData@Heart consortium resulted in a total of 56 participating datasets relating to 28 PIs of 13 different institutes, organizations or companies. PIs were identified and contacted for retrieval of documents. Out of 28 PIs we addressed by e-mail and/or telephone, 20 (71%) responded covering a total of 31/56 (55%) datasets. Eight datasets were removed from the list because they did not contribute patient-level data to BigData@Heart.

For 24/48 (50%) datasets we were sent some form of documentation or reference to conditions for data sharing. For 17/24 datasets actual documentation was provided. For the other 7 datasets a statement was received by e-mail with a reference to policy or legislation. Our team received 60 documents consisting of: 26 informed consent forms (often including patient information), 15 patient information forms, 8 data transfer and data access agreements (DTAs/DAAs), 4 study protocols, 3 blank case report forms (CRFs) of clinical trials, 2 policy statements and 2 questionnaires for participants. After initial screening, blank CRFs and questionnaires were excluded since there were no conditions referred to in these documents, leaving 55 documents for further analysis.

### Analysis of prespecified conditions for data sharing

We extracted information from the 55 identified documents along the lines of the following five key elements: statements on data sharing, purpose limitation, level of de-identification, terms of issuance and reference to ‘policy otherwise’. An overview of the conditions for data sharing as stated in the received ethico-legal documentation from 17 datasets can be found in the Additional file 1: Table S1.

The documentation on most datasets included a statement about data sharing. Statements about data sharing were more common among non-experimental, non-commercial studies (cohorts) than among clinical trials performed by industry. For all clinical trials, no explicit mentioning of data sharing for future health research was found. In most documents data sharing was described as the “conditional” sharing of the data collected during the study with “third parties”. Conditions and third parties were not further specified in a number of documents.

### Purpose limitation

For *use of stored data*, most datasets restrict the permitted use to “scientific research”. For such studies, some patient information forms mention that it “might be necessary to work with commercial companies”. One document states that data will never be sold to commercial companies. Another declares that the database is established by a non-profit organization, but that the results from collaboration with a commercial company may become property of that company and may be exploited for commercial purposes. Patients are reminded that they have no claim to property rights in such cases. Most document pertaining to observational studies/registries restrict use of stored data to scientific research within the scope of the primary research activities only, either by limiting use to disease area (e.g., cardiovascular disease) or “relevance” to the dataset or original study itself. For most datasets, it appears that use of stored data is limited to the questions specified in a pre-determined research plan that is submitted for approval by the primary study team. Some industry-sponsored trials state that property rights may be shared or transferred to another sponsor/owner (without specifying use).

### Level of de-identification

Many documents explicitly mention “coded data” as a condition for data sharing. The key to access to the directly identifiable personal data is described to remain with either 1) only the research team, 2) only one researcher from the team 3) or only the treating clinician. Coded data is mostly described in informed consent and patient information forms as “you will only be identified by a number” or “you will be given a special code that identifies you”. According to different informed consent documents, use of “coded data” may indicate either “use without revealing your identity”, that “your identification will be removed”, “that it is unlikely that anyone will be able to identify you”, that “you cannot be recognized by it (removal of full name and address)” or that “your data is anonymous”.

Industry-sponsored trials sometimes state that: “all personal data that leaves your doctor’s site will be anonymized/in anonymous form”. In documents where *data sharing with third parties* is explicitly addressed, various (descriptions of) levels of de-identification are mentioned. For example, some state that “your data will be shared in such a way that the data cannot be traced back to you”, that “data is issued with unique pseudonyms as patient identifiers” or that “requested data have been made (fully) anonymous”. In some cases, where “full anonymization” is not considered possible, data is “de-identified to the fullest extent possible to ensure data is unidentifiable”. Only two clinical trials explicitly mention which personal identifiers will be removed in order to de-identify the data. Some datasets only allow sharing of aggregate data, not individualized data. One dataset mentions that, in some instances, sharing of personal data is unavoidable, and that a separate processing agreement will need to be signed.

### Terms of issuance

For *use of stored data*, most datasets require interested users to submit a formal request to the original study team. In many cases, data transfer agreements (DTAs) are used to bind users to terms and conditions. General templates are used as well as specific DTAs issued per project. All DTAs mention the required level of de-identification and state that users are not permitted to re-identify patients or share the data with persons other than those directly working on the specific research project, for which approval was granted. In a number of DTAs, use of the data for purposes other than the research objectives outlined in the application is prohibited. Many research teams also specify conditions with respect to publication of the results generated from the data. According to a few DTAs, users have to agree with someone from the original team being involved in the study, for example, in the analyses or as an author of the publication. Often, DTAs will include responsibilities with respect to data security. Sometimes this responsibility is placed on the user, sometimes on the provider and sometimes on both. Only one dataset requires express written informed consent from the data subjects for secondary use.

### Compliance with ‘policy otherwise’

Most DTAs include a paragraph that refers to compliance with what we will call ‘policy otherwise’. Here, it is relevant to note that the GDPR only came into force as of 25 May 2018, meaning that reviewed documents will likely not refer to this regulation. The vast majority of transfer agreements do make reference to national legislation. A general statement often encountered in DTAs is that data will be used in accordance with “all the applicable local laws, regulations, statutes and guidelines which are applicable to the recipient’s use of the data”. In informed consent forms, reference is made more generally to legislation (“data is kept confidential within the limits of the law”). For international clinical trials, sponsors often mention in patient information forms that participants “should be aware that some countries may not offer the same level of privacy protection as [they] are used to in the country where [they] live or where this study is conducted”. Two datasets refer to their own institute-specific/study-specific privacy regulations.

## Discussion

For this exploratory study, we reviewed 55 dataset-specific ethico-legal documents for conditions for data sharing and data access within BigData@Heart. We observed convergence on the conditions that data sharing is only permitted for scientific research, in anonymized, or else, coded form, after approval from a designated committee (steering, ethics, or the original research team granting approval), with the appropriate measures for data security in place and in compliance with the laws and regulations that are applicable. Despite consensus on these four fairly general conditions for data sharing, we foresee particular challenges and outline how prespecified conditions could inform our thinking about an ethical governance framework for future data sharing initiatives.

### Prespecified conditions as challenges

The condition that data may only be shared for scientific research is problematic in its open formulation. Even ‘broad informed consent’ places certain limitations on the future use of health data and requires specification of, among others, foreseeable uses, intended goals of such use, whether only for basic or applied research, or also for commercial purposes [18]. For example, while some documents explicitly mention whether scientific research for commercial purposes is permitted, others do not. Commentators have already noted that obtaining prospective, meaningful informed consent is becoming virtually impossible [19]. This has caused a trend to argue for moving away from consent as the standard legal basis for the use of personal data in health research. For data collection for which consent has already been obtained, data sharing initiatives should address the question: what kinds of research questions, which areas of research and what motives are acceptable (See Table 1)? For prospective data collection, raising awareness among researchers about how to determine the purpose limitation should become a priority.

**Table 1:** Challenges and action points for data sharing initiatives resulting from prespecified conditions

Condition	Challenge	Action point
Data sharing is only allowed for …		
Scientific research	What kinds of questions, areas of research and motives are acceptable?	Establish the scope of scientific research questions that may be addressed
In anonymized or coded form	In case full anonymity cannot be guaranteed, can data still be shared? If yes, on what grounds and how?	Establish how to deal with varying levels of de-identification
After approval from a review committee and with acceptance of security measures	When and how should review committees operate in the context of Big Data-driven research platforms?	Establish the role and responsibilities of review committees
In compliance with applicable laws, rules and regulations	Laws, rules and regulations may offer different interpretations of data sharing	Establish what laws, rules and regulations mean by data sharing
–	Documents may permit “conditional data sharing with third parties” without specifying these conditions	Establish how to deal with datasets that do not have a system in place for (international) data sharing

Second, “anonymized” and “coded” are terms that are used interchangeably. In the documents we reviewed, it appeared that different descriptions are attached to the same terms, that the same description is used for different terms, that different terms may be used, and that sometimes terms are not properly defined (See Table 2). This is particularly relevant from a legal point of view, since the GDPR only applies to personal data. It is important to note here that under the GDPR coded or pseudonymized data are considered personal data. Anonymized data does not enjoy legal protection from the GDPR. We anticipate that data from electronic health records and research databases will most probably have to be dealt with as personal data. One of our concerns is that informed consent documents might promise a higher level of de-identification than practically achievable or legally required. Researchers are in principle bound to such promises made during the informed consent process. Data sharing initiatives will then need to establish how to deal with varying levels of de-identification while considering alternative strategies to safeguard privacy and confidentiality.

**Table 2:** Various descriptions of the level of de-identification as encountered in the reviewed documents

“your data is anonymous”	
“requested data have been made (fully) anonymous”	
“you cannot be recognized by it (removal of full name and address)”	
“your data will be shared in such a way that the data cannot be traced back to you”	
“your identification will be removed”	
“use without revealing your identity”	
“that it is unlikely that anyone will be able to identify you”	
“identification of the individual is not reasonably possible”	
“if full anonymization is not possible, data is de-identified to the fullest extent possible to ensure data is unidentifiable”	
“you will only be identified by a number”	
“you will be given a special code that identifies you”	
“data is issued without directly identifiable patient numbers”	
“data is issued with unique pseudonyms as patient identifiers”	

Third, obtaining approval from local committees or research teams and reaching agreements about academic acknowledgements may pose great logistical and practical challenges for large-scale data sharing for international health research. In practice, we expect that local committees and research teams are currently doing most of the work to ensure responsible use (approval per request, evaluation of research plan, by issuing DTAs, etc.). Patients and healthy individuals who have agreed (either through opt-in or opt-out) to have their data stored for future use most probably place great trust in the evaluation process of new requests [14, 16]. This condition raises questions of when and how review committees should operate in the context of Big Data-driven research platforms.

Also, use of the term “data sharing” in itself is not unambiguous. Many policy and legal documents include statements about data sharing; however, data sharing may in practice refer to different activities. For example, it may refer to the mode of sharing (could entail physically transferring data to users versus distributed access), access and usage (viewing only versus performing analyses) or scope (current study versus future studies). In such cases, understanding what these policies and regulations actually mean by “data sharing” will be important.

### How prespecified conditions could inform ethical governance

Prespecified conditions tied to original datasets can inform our thinking about an ethical governance framework for data sharing platforms in three ways. First, agreement on the four conditions suggests what could be considered the moral fundaments of responsible data sharing. Second, an ethical governance framework will need to address the challenges identified with open, ambiguous or restrictive conditions. Third, omissions or “white spots” in these source documents point towards where additional ethical guidance is needed. A striking observation is that many documents are silent on a number of ethical conditions for data sharing as specified in international guidelines and declarations. For example, the Council for International Organizations of Medical Sciences’ (CIOMS) *International Ethical Guidelines for Health-related Research Involving Humans* state that if stored data is to be used for multiple and indefinite uses, consent is only valid if the concerned individuals have been adequately informed about, among others, the procedures for return of results, including incidental findings, and how to retract authorization for further research [18]. Other items that were often missing in reviewed documents were information about intellectual property issues and the transfer of data to other institutions or third countries [20]. But above all, there is the question of how to deal with datasets that have no system or conditions in place for data sharing. An ethical governance framework stipulating the conditions for responsible data sharing will be all the more important for such datasets.

We hasten to say that from this observation it does not follow that such conditions do not apply to the considered datasets. It simply means we did not encounter conditions of the like in the documents we were sent. We suggest that such omissions could imply conditions that are either more or less restrictive than, for example, the GDPR. Not knowing the conditions practically used (as opposed to literally defined) is a potential shortcoming of this case study. Another limitation is that DTAs are mostly written as standard templates, and paragraphs may be altered depending on specific requester-provider interactions. Concerning informed consent forms, different versions may be issued over the years – especially in longitudinal cohorts – and we did not specifically ask for all these different versions. This means that our collection of documents is by no means exhaustive or comprehensive. Nevertheless, our results do reflect the conditions as specified in a representative sample of ethico-legal documents.

## Conclusions

Responsible data sharing in health research entails more than compliance with the GDPR. Data sharing specifications developed at local European research sites need to be taken into account when designing complementary ethical governance for Big Data-driven translational research platforms. From the BigData@Heart platform, we have learned that a governance system, however, also cannot only fall back on locally devised policies and conditions. They serve as a vital starting point but are clearly not devised with the prospect of Big Data research in mind. There is an evident need to reconcile these issues in a new and adaptable governance framework for platforms such as BigData@Heart. At this stage, concrete steps for data sharing initiatives to concentrate on are: (1) the scope of the research questions that may be addressed, (2) how to deal with varying levels and requirements of de-identification, (3) determining the role and responsibilities of review committees, (4) establishing what policies and regulations mean by “data sharing” and (5) how to deal with datasets that have no system in place for data sharing.

## Supplementary information

**Additional file 1: Table S1.** Overview of conditions for data sharing as stated in received ethico-legal documentation.

## Data Availability

All data generated during this study are included in this published article. For access to analyzed source documents the corresponding author will enquire about permissions among participating study teams within BigData@Heart, upon reasonable request.

## Abbreviations

CIOMS: Council for International Organizations of Medical Sciences
CRF: Case record form
DAA: Data access agreement
DoA: Description of action
DTA: Data transfer agreement
EHR: Electronic health record
GDPR: General Data Protection Regulation
PI: Principal Investigator
