# Supplementary material for: Responsible data sharing in a big data-driven translational research platform: lessons learned
Source: BMC Med Inform Decis Mak. 2019 Dec 30;19:283. doi: 10.1186/s12911-019-1001-y (PMC6936121; doi:10.1186/s12911-019-1001-y)
Supplement: Supplementary file 1 — Additional file 1: Table S1. Overview of conditions for data sharing as stated in received ethico-legal documentation. [file 12911_2019_1001_MOESM1_ESM.docx]

**Table S1.** Overview of conditions for data-sharing as stated in received ethico-legal documentation.

| **No.** | **Statement on data-sharing** | **Purpose limitation** | **Level of de-identification** | **Issuance terms** | **Reference to ‘policy otherwise’** |
| --- | --- | --- | --- | --- | --- |
| 1 | Data may be shared conditionally with third parties  Consent to re-contact for additional data collection for future studies | Scientific research purposes  *Current:* “For this study it may be necessary to share data with third parties (institutes or companies).” | Data are coded  “Your data will be stored in coded form”  “Only the PI and those persons directly related to the research have access to the key to your personal data” | Per query approval for secondary use required from scientific advice committee of primary research site. | - |
| 2 | Data may be shared conditionally with third parties  Consent to re-contact for additional data collection for future studies and communication of incidental findings. | Scientific research in cardiovascular disease  *Current:* “For this study it may be necessary to share data with third parties (institutes or companies).” | Data are coded  “Your data will be stored in coded form.”  “Only the researcher has access to the key to your personal data” | Per query approval for secondary use required from scientific advice committee of primary research site.  “Data will never be sold to companies” | - |
| 3 | None described in consent and patient information forms.  DTA suggests sharing is allowed.  Consent to re-contact for additional data collection for future studies | Scientific research purposes  “If the researcher in the future wants to use the data generated by the researcher in our samples for research questions other than the ones here defined, a new agreement has to be reached” | Data is anonymized  DTA: “requested data have been made **anonymous**” | Anonymized data issued per query by primary research team  Recipient is responsible for data safety during use.  Analysis of the data is only allowed in collaboration with the primary research team. | “The researchers will treat my data confidentially and will comply with the rules as laid out in the *Wet Persoonsregitraties* (Dutch Personal registries Act) and the Privacy Regulations of the Department of Epidemiology” |
| 4 | Data-sharing permitted through data access and data use agreements between scientific data broker and providers and users of data | Scientific research purposes  Broker “supports scientific research projects by linking data sets owned/controlled by third party data users” and by providing access per query to interested users for their research | May contain personal data  “Broker may receive **personal data** from user” | Agreement to terms for secondary use as laid out in data access and data use contracts between broker, provider and user.  “No re-use or use other than permitted use”  “User has the right to disclose the provided data to other participants in the research for use within the same research only” (user must make sure a written agreement is signed that binds participants to the same terms)  In case broker needs to process personal data, broker and user will enter into a separate data processing agreement.  DPA: “Broker has implemented appropriate technical and organizational measures to protect personal data against accidental or unlawful destruction or accidental loss, alteration, unauthorized disclosure or access.”  User shall not attempt to re-identify or contact individuals with the personal data. | Data access agreement is “governed by the laws of the Netherlands”  Data should be used in compliance with Dutch Personal Data Protection Act. |
| 5 | None about data-sharing  Consent to re-contact for additional data collection for future studies | Scientific research in cardiovascular disease | Data are coded  “Your data will be treated confidentially and will be stored using a number.” | Per query approval for secondary use required from scientific advice committee of primary research site. | - |
| 6 | Data may be shared conditionally with third parties  Consent to re-contact for future studies | For future scientific research  To treating general practitioners for medical care purposes  To municipal health authorities  To the individual participant on request | Data is coded for primary research activities  Data is anonymized for secondary use by third parties  “Database to be issued for future studies is stripped from data in such a way and at the aggregated level that **identification of the individual is not reasonably possible**” | Only researcher and treating GP have access to key to personal data  Anonymized data issued at the aggregated level per query by primary research site.  Recipient is responsible for data safety during use.  Secondary research activities must include member of the original project team. | Reference to study-specific privacy regulations |
| 7 | Data-sharing with third parties only permitted after written informed consent of participant  Consent to re-contact for additional data collection for future studies and communication of incidental findings. | Scientific research  “I consent to storage of my data for future (coded) use for scientific research as mentioned in this information letter” | Data are coded for use by primary research site  Data are anonymized for secondary use by third parties | Only after written informed consent from participant  Use of DTA  No use for commercial purposes  Only research involving the primary research site | - |
| 8 | Data-sharing permitted within the same academic medical centre  This dataset comprises of “electronically registered care data of patients treated in our centre that is made accessible for scientific research. Researchers within our centre may submit a request to our data management team to use the database.” | Scientific research that is relevant to the primary research activities | Data is coded  “Data is always **issued to user without directly identifiable patient numbers**”  “Per project **unique pseudonyms** are issued as patient identifiers” | Issuance of coded data per query after evaluation by the primary investigators (relevance to primary research, scientific validity, feasibility)  Secondary research activities must include member of the original project team. | - |
| 9 | Data may be conditionally shared with third companies (including companies) | Non-commercial research purposes with academic hospitals and research institutes  Commercial purposes with companies  “Results of collaboration with companies may become property of those companies. All research results, including future commercial developments, will benefit health care. You cannot derive property rights from those results. You may choose not to provide your data and material for commercial research purposes.” | Data are anonymised/coded (?)  “Your data will be stored in coded form”  “For the purposes of this study, it may be necessary to share data with other academic hospitals or companies. This will always occur in such a way that the **data cannot be traced back to you**.”  DTA: Data is fully **anonymised**, and if not possible, “**de-identified to the fullest extent possible (…) to ensure all records are unidentifiable**” | Secondary use permitted through DTA between user and provider.  In principle data is anonymized, otherwise “to the fullest extent possible”  No efforts may be undertaken by the user to re-identify participants.  Both provider and user are responsible for appropriate data safety measures.  No sharing permitted beyond intended use | - |
| 10 | No explicit mentioning of data sharing for secondary use | “The results from this study may be published in medical journals, used in scientific report or presented to the regulatory authorities as part of an application by the sponsor to market drug [X] for disease [Y]” | Data is coded (?)  “All information about you that leaves the hospital will have your full address and name removed so that **you cannot be recognised** from it.”  “You will be **identified by a number** and the only person who will be able to link this number to your name is your hospital doctor.” | - | “Any transfer of data will take place in compliance with the regulations protecting the processing and transfer of personal data” |
| 11 | No explicit mentioning of data sharing for secondary use | “The data and results from this study may be published in medical journals or used in scientific reports, but your name will never appear.” | Data is anonymised  “All personal data collected during the study will be dealt with confidentially. It will be used only for the purpose of the research and for submission to competent authorities in **an anonymous form.”**  “All information about you that leaves your doctor’s office will be **anonymised**” | - | “Any transfer of data will take place in compliance with the regulations protecting the processing and transfer of personal data” |
| 12 | No explicit mentioning of data sharing for secondary use | “The data and results from this study may be published in medical journals or used in scientific reports, but your name will never appear.” | Data is anonymized  “All personal data collected during the study will be dealt with confidentially. It will be used only for the purpose of the research and for submission to competent authorities in an **anonymous form.”**  “All information about you that leaves your doctor’s office will be **anonymous**” | - | “Any transfer of data will take place in compliance with the regulations protecting the processing and transfer of personal data” |
| 23 | No explicit mentioning of data sharing for secondary use | “The data and results from this study may be published in medical journals or used in scientific reports, but your name will never appear.” | Data is anonymized/coded (?)  “All personal data collected during the study will be dealt with confidentially. It will be used only for the purpose of the research and for submission to competent authorities in an **anonymous form** (**you will be identified only by a number**).”  “All information about you that leaves your doctor’s site will be **anonymised** (**you will be identified only by a number**)” | - | “Any transfer of data will take place in compliance with the regulations protecting the processing and transfer of personal data” |
| 14 | No explicit mentioning of data sharing for secondary use | “The data and results from this study may be published in medical journals or used in scientific reports, but your name will never appear.” | Data is anonymised/coded (?)  “All personal data collected during the study will be dealt with confidentially. It will be used only for the purpose of the research and for submission to competent authorities in an **anonymous form** (**you will be identified only by a number**).”  “All information about you that leaves your doctor’s site will be **anonymised** (**you will be identified only by a number**)” | - | “Any transfer of data will take place in compliance with the regulations protecting the processing and transfer of personal data” |
| 15 | No explicit mentioning of data sharing for secondary use | “The data and results from this study may be published in medical journals or used in scientific reports, but your name will never appear.” | Data is coded  “All personal data collected during the study will be dealt with strict confidentiality. It will be used only for the purpose of the research and for submission to competent authorities **without revealing your identity** (**you will be identified only by a number**)”  “All information about you collected for the research which leaves your research doctor's site or that will be transmitted by your ICM to Medtronic database will have **your identification removed** (**you will be identified only by a number**).” | - | “Any transfer of data will take place in compliance with the regulations protecting the processing and transfer of personal data” |
| 16 | Data may be shared conditionally with third parties (including companies) | *Current:* “The sponsor may share your coded information, as necessary, with other members of the sponsor’s worldwide group of related companies, people and companies who work with the sponsor and who work within the scope of this consent.”  *Future:* “In the future, the sponsor may pass or share its’ ownership rights to this trial and study drug to another company. In such a case, the **coded study information will be transferred to the new sponsor**.” | Data is coded  “Your name will be replaced with a **special code that identifies you**. This code will be used by the study sponsor and their representatives for the study purposes mentioned here. | - | “The study information will be kept confidential within the limits of the law.”  “You should be aware that some countries may not offer the same level of privacy protection as you are used to in the country where you live or where this study is conducted.” |
| 17 | Data may be shared conditionally with third parties (including companies) | *Current:* “Within the scope of this consent, the sponsor may share your age/year of birth (and date of birth, if allowed), gender, as well as your code, study information, and biological samples as necessary, with other members of the sponsor’s worldwide group of related companies, people and companies who have a contract and work with the sponsor, and other third parties (which may include third parties in other jurisdictions)”  *Future:* “In the future, the sponsor may pass or share its ownership rights to this study and the study treatment to another company. In such a case, **your personal data will be transferred to the new owner**.” | Data is coded  “The study doctor will replace your name and other general information about you, excluding age/year of birth (and date of birth if allowed) and gender, with a **special code that identifies you**.”  “The study doctor will associate the code with the study information and biological samples to make it **unlikely that anyone will be able to identify you**.” | - | “The study information will be kept confidential within the limits of the law.”  “You should be aware that some countries may not offer the same level of privacy protection as you are used to in the country where you live or where this study is conducted.” |
| 18 & 19 | Data may be shared conditionally with third parties | Not specified | Not specified | Approval per query  DTAs issued per project  “Any project proposal needs approval by the registry steering committee. Thereafter ethics approval or amendment to existing ethics application.”  “In Swedish registries patients do not provide written informed consent, but are informed of entry into national registries. They are allowed to opt out at any time.”  “No general data transfer agreements exist, these are issued per project.” | Reference to Swedish data protection law |
| 20-24 | Data may be shared conditionally with third parties | Not specified | Not specified | Approval per query  Only aggregated data may be shared  Analyses are performed in-house upon request | Reference to German data protection laws  “Permission to share is not specifically captured in the informed consent forms, but on the agreement policy of our studies” |
